# Effects of resilience on impulsivity, cognition and depression during protracted withdrawal among Chinese male methamphetamine users

**DOI:** 10.1186/s12888-022-04041-8

**Published:** 2022-06-21

**Authors:** He He, Siyao Zhou, Chenhui Peng, Wang Ran, Siyu Tong, Lan Hong, Fangfang Cai, Wei Jin, Yile Jiang, Mengjia Li, Xuanping Wang, Mengdan Luo, Wei Wang, Ke Zhao

**Affiliations:** 1 School of Mental Health, Wenzhou Medical University, Chashan Higher Education Park, Ouhai District, Wenzhou, 325000 China; 2 The Second Affiliated Hospital, College of Medicine, Zhejiang University, Hangzhou, China; 3 The Affiliated Kangning Hospital of Wenzhou Medical University, Wenzhou, China; 4 School of Mental Health, Key Laboratory of Alzheimer’s Disease of Zhejiang Province, Wenzhou Medical University, Wenzhou, China

**Keywords:** Methamphetamine, Resilience, Impulsivity, Cognitive function, Depression

## Abstract

**Background:**

Methamphetamine (METH) dependence is a complex and dynamic public health problem. Long-term abuse of METH can increase numerous risks of mental and physical problems. Currently, the methods to reduce METH dependence and improve the withdrawal symptoms are limited and ineffective. Resilience is seen as a multidimensional and dynamic capability to recover or bounce back from stressful events and is also generally considered as a protective factor against mental dysfunction.

**Methods:**

One hundred thirty-four males with METH dependence were consecutively recruited from Huanglong Compulsory Isolated Detoxification Center between 2019 and 2021, of whom 112 were into the group. The Connor-Davidson Resilience Scale (CD-RISC), Self-rating depression scale (SDS), Self-rating anxiety scale (SAS), Barratt Impulsiveness Scale-11(BIS-11), and the Repeatable Battery for the Assessment of Neuropsychological Status (Rbans) were used to evaluate resilience, depression, anxiety, impulsivity, and cognition respectively.

**Results:**

The results mainly indicated that high resilience group showed lower SDS, SAS and BIS-11 scores than low resilience group (all *p* < 0.05). Besides, the total scores of Rbans were higher in high resilience groups than low resilience group (both *p* < 0.05). Moreover, linear regression results showed that resilience may be influenced by the scores of SDS and SAS.

**Conclusions:**

Resilience is negatively correlated with impulsivity and depression. Besides, it is also positively associated with cognitive function. Drug users with higher resilience may have a strong ability to mobilize psychological resources to create a good psychological environment, which may have a positive effect on the relief or improvement of symptoms.

**Supplementary Information:**

The online version contains supplementary material available at 10.1186/s12888-022-04041-8.

## Introduction

According to 2020 World Drug Report, approximately 27 million people are estimated to have used amphetamines worldwide in the past year. The number of people using amphetamine, especially methamphetamine (METH), is increasing in parts of Asia and North America [1]. METH addiction is a biological psychosocial disorder that remains a global complex and dynamic public health problem [2]. In China, there is an increasing number of individuals addicted to METH [3]. So far, the methods to reduce METH dependence, improve the symptoms caused by METH use, and prevent relapse are limited [4, 5], which include both pharmacological and non-pharmacological treatments. There are no specific pharmacological treatments for METH dependence [6], so non-pharmacological treatments are becoming increasingly important in intervention options. Non-pharmacological treatments considered effective in METH dependence mainly include cognitive-behavior therapy (CBT), motivational interviewing (MI) and/or contingency management (CM) [7, 8].

Mental excitement, hypersexuality, agitation and violent behavior are common effects of high-dose METH. Long-term use of METH can produce strong dependence and, once the drug is stopped, withdrawal symptoms will appear. During METH dependence withdrawal, patients may experience intense drug cravings [9], depression [10], anxiety [11], fatigue [12], and mental illness [13]. If the withdrawal symptoms are not well treated, they may cause more severe problems [14]. Increasing evidence showed that METH-dependent patients experience impaired cognitive functions (i.e., executive function, attention, social cognition, and working memory) [15]. These deficiencies may partially lead to persistent drug use and poor or unhealthy decision-making [16]. Cognitive defects can also cause behavioral changes, associated with high levels of impulsiveness, hostility and aggression [17, 18]. Desey Tziortzis et al. reported a marginal relationship between impulsivity and craving [19], suggesting the degree of impulsivity can positively predict the probability of relapse. Research has also shown that people who abuse METH for a long time often experience depressive symptoms, especially during METH withdrawal [20]. It is common for depression and substance use to occur at the same time [21]. Some researchers support pharmacological approaches to treat METH use or withdrawal and/or depression, while others favour non-pharmacological therapies, such as CBT and mindfulness therapy [22]. The effectiveness of pharmacological interventions to treat METH addiction and the resulting symptoms seems to be limited [23, 24]. Most common intervention strategies still focus on non-pharmacological treatment.

Previous studies suggested that early stressful life events are important risk factors for alcohol and illicit substances addiction [25]. However, not all responses to stress are bad, as some stressors may mobilize resilient attempts to protect individuals from adverse factors [26]. Resilience is the flexible use of psychological resources for adapting to adversity [27]. Dyer and McGuinness indicated that resilience describes a process whereby people bounce back from adversity and go on with their lives [28], which may be considered as an outcome-oriented or a process-oriented approach. It is a dynamic process highly influenced by protective factors, which consists of internal and external factors. The internal factors include genetics or epi-genetics and personality traits (e.g., optimism, tenacity), or beliefs (e.g., self-efficacy) [29] and the external factors include social support or socioeconomic status [30]. In this vein, resilience can lead to a stable psychological state during or after an adversity, or a temporary pattern of disturbance followed by a relatively rapid and successful recovery. However, some researchers have regarded resilience as a trait-oriented approach characterized by tenacity, which is associated with three primary attributes: a greater sense of control over their lives, commitment to the areas of their life even when experiencing stressors, and a perception that change is a challenge rather than a threat [31]. When we study the role of resilience, we tend to regard it as an outcome-oriented or a process-oriented way to explore the dynamic process, because resilience can be influenced by many factors.

Some studies also highlighted the role of resilience in the prevention of substance addiction. For example, Dullius et al. suggested resilience played an important role in moderating stress and negative emotions in patients with alcohol dependence [32]. And low resilience was reported to be related to alcohol or drug problems and poor working memory performance [33]. Furthermore, a study suggested brain networks in patients with drug addiction showed impaired goal-oriented actions were associated with the resilience system related to behavior regulation [34]. Since resilience is the flexible adaptability of dealing with stress-related mental diseases, we sought to explore the connection between resilience and METH addiction during protracted withdrawal to provide psychotherapy suggestions.

The level of resilience may be related to cognition, depression levels and the degree of impulsivity. The hypothesis assessed during the current study is that the improvement of resilience levels positively affects the improvement of the above factors.

## Methods

### Participants

**Fig. 1:**
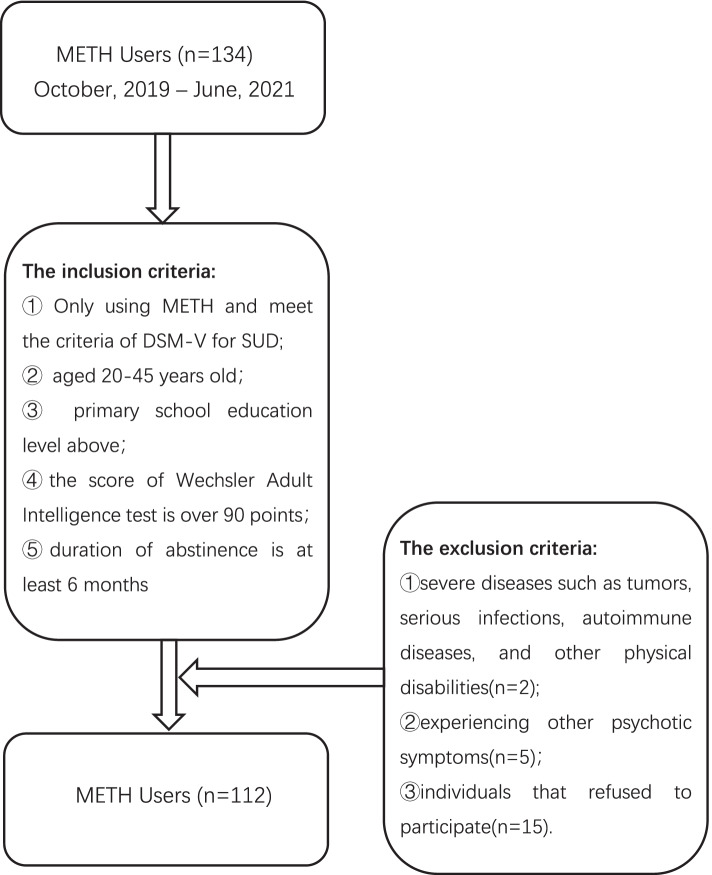
Sample flow chart. Note: METH Methamphetamine; SUD substance use disorder

One hundred thirty-four male individuals were recruited from the Huanglong Compulsory Isolated Detoxification Center in Wenzhou between October 2019 and June 2021, of whom 112 met the inclusion criteria in this study. The inclusion criteria included: (1) with a history of only using METH and fulfill the criteria of Diagnostic and Statistical Manual of Mental Disorders 5th edition (DSM-V) for Stimulant Use Disorder (SUD);(2) Han ethnicity, aged between 20 and 45 years old;(3) primary school education level above;(4) the score of Wechsler Adult Intelligence test is over 90 points;(5) duration of abstinence is at least 6 months. And the exclusion criteria were:(1) severe diseases such as tumors, serious infections, autoimmune diseases, and other physical disabilities; (2) experiencing other psychotic symptoms, and (3) individuals that refused to participate. Detailed information about the participants is presented in Fig. 1.

The enrolled individuals were assessed from high to low according to the scores of the Connor-Davidson Resilience Scale. The top 33% of subjects were assigned to the high resilience group (*n* = 37), the bottom 33% of the subjects were assigned to the low resilience group (*n* = 37), and the remaining subjects were assigned to the medium resilience group (*n* = 38).

### Collection and evaluation of socio-demographic and clinical measures

The socio-demographic data included age, education, living styles, marital status, religious belief and duration of drug exposure. The clinical measures included the Self-Rating Depression Scale (SDS), the Self-Rating Anxiety Scale (SAS), the Barratt Impulsiveness Scale-11(BIS-11), the Repeatable Battery for the Assessment of Neuropsychological Status (RBANS), the Connor-Davidson Resilience Scale (CD-RISC), and the Somatic Self-Rating Scale (SSS).

SDS is a self-rated scale to evaluate the severity of depression [35], and consists of 20 questions. The standard score is calculated by summing all question scores, and the total score is 1.25 times the standard score, using an integral part. The higher the score correlates to more severe depression. Cronbach’s α in the present study was 0.83.

SAS is also a self-rated scale, which is used to assess the degree of anxiety. It consists of 20 items on a 1–4 Likert-type scale. A higher score suggests more severe anxiety [36]. Cronbach’s α in the present study was 0.81.

The BIS-11 scoring consists of 30 items on a four-point Likert scale (1 = “rarely/never” to 4 = “almost always/always”), which is used to measure impulsivity [37]. The scales can be divided into three dimensions (cognitive, motor, and non-planned impulsivity). The higher scores of each scale suggest the higher impulsivity levels shown by individuals. Cronbach’s α in the present study was 0.79.

RBANS scoring comprises 12 subtests that yield five index scores (immediate memory, visuospatial, language, attention, and delayed memory) and one total score. The total scale score of RBANS can be used to evaluate cognitive function, and the higher the total scale score, the better the cognitive function [38]. Cronbach’s α in the present study was 0.84.

CD-RISC is a self-reported 25-item scale, which is used to evaluate the resilience. The CD-RISC developed by Connor and Davidson had a five-factor structure by using exploratory factor analysis (EFA), which included hardness, persistence, optimism, support, and spirituality [39]. However, neither the confirmatory factor analysis nor the explorative factor analysis on the data from a relatively large sample of Chinese people could replicate the American 5-factor structure of resilience. In 2007, Yu et al. modified the scale and contains three factors: tenacity, strength, and optimism [40]. The scale employs 5-point Likert-type ratings ranging from 0 (not true at all) to 4 (true all the time). It focuses on assessing different resilience factors to maintain or regain mental health. Generally, higher scores reflect greater resilience. Cronbach’s α in the present study was 0.82.

The SSS includes 20 items, with each item scored on a 4-point scale (1, none; 2, slight; 3, moderate; and 4, marked). There were 9 factors about somatization symptom, 5 factors about anxiety symptom, 4 factors about depression symptom and 2 factors about anxiety and depression symptom. Generally, the higher the score, the more severe the somatization symptoms. This scale is specifically designed to check for possible emotional problems and corresponding somatization symptoms during your onset, and if the selected items have a score greater than 30, patients need to consider medical treatment [41, 42]. Cronbach’s α in the present study was 0.78.

### Statistical analysis

Normal distribution was assessed by the Shapiro–Wilk test. The socio-demographic and clinical characteristics of the low resilience, moderate resilience and high resilience groups were compared using the one-way analysis of variance (ANOVA) for continuous variables and the chi-square test for categorical variables. Homogeneity of variances test and between-subjects effects test were used. Besides, we also performed post hoc pairwise comparisons. In addition, the correlations among three dimensions of CD-RISC and SDS, SAS, BIS-11 and Rbans were also evaluated. Bonferroni corrections were applied to adjust for multiple testing. Further, multiple linear regression was used to evaluate the risk factors for resilience during protracted METH withdrawal. All data analysis was calculated with SPSS version 25.0 with two-sided *p* values of 0.05.

## Results

The socio-demographics and the clinical characteristics of the three groups were summarized in Table 1. The data was normally distributed (the result of Shapiro–Wilk test was listed in Supplemental Table 1). Table 1 showed that there was no significant difference among the groups in age, education, living styles, marital status, religious belief, the duration of drug exposure as well as the scores of SSS. Further, the main results revealed that high and medium resilience groups showed significantly lower SDS scores compared with the low resilience group (both *p* < 0.05). Participants with high resilience also reported lower scores of SAS compared with those with low resilience (*p* < 0.05). Notably, the high resilience group showed lower cognitive impulsiveness, non-planning impulsiveness and BIS-11 total scores than low resilience group (all *p* < 0.05). Besides, compared with low resilience group, the delayed memory was better in high resilience group (*p* < 0.05) and attention was better in the medium resilience group (*p* < 0.05). The total scores of Rbans were also higher in high and medium resilience groups than low resilience group (both *p* < 0.05). Homogeneity of variances test was checked (all *p* > 0.05, Supplemental Table 2) and the reliability of the relationship was good (all *p* > 0.05, Supplemental Table 3).

**Table 1:** Socio-demographics and clinical characteristics among the groups of low, medium and high resilience

	Low resilience (*n* = 37)	Medium resilience (*n* = 38)	High resilience (*n* = 37)	Overall *P*-value	Post hoc comparisons
Age, mean (SD)	35.72(5.54)	35.92(6.29)	35.40 (5.68)	0.951	
**Education**				0.553	
primary school education level, n (%)	5(13%)	2(4%)	5(13%)		
Junior high school education level, n (%)	25(67%)	25(67%)	21(57%)		
Senior high school education level, n (%)	7(20%)	11(29%)	11(30%)		
**Living styles**				0.840	
Live with family	25(67%)	24(64%)	25(67%)		
Live without family	12(33%)	14(36%)	12(33%)		
**Marital status**				0.424	
Married	20(53%)	17(44%)	16(44%)		
Not married	13(34%)	11(29%)	15(40%)		
Divorced	4(13%)	10(27%)	6(16%)		
**Religious belief**				0.349	
Yes	21(56%)	17(44%)	15(40%)		
No	16(44%)	21(56%)	22(60%)		
Duration of drug exposure, years, mean (SD)	13.72(6.32)	12.92(6.70)	14.56(7.52)	0.701	
SDS, mean (SD)	55.76(7.28)	47.63(9.44)	40.04(8.48)	< 0.001***	1 > 2**; 1 > 3***; 2 > 3**
SAS, mean (SD)	42.84(7.39)	42.60(6.51)	38.28(7.40)	0.045*	1 > 3*; 2 > 3*
SSS, mean (SD)	28.04(7.99)	29.40(8.42)	26.44(6.12)	0.388	
BIS-11					
cognitive impulsiveness, mean (SD)	30.94(5.70)	28.57(3.10)	26.26(5.04)	0.004**	1 > 3**
motor impulsiveness, mean (SD)	21.28(8.73)	24.27(6.40)	20.57(6.85)	0.179	
non-planning impulsiveness, mean (SD)	32.05(7.46)	28.42(6.47)	25.42(8.03)	0.009**	1 > 3**
Scale total scores, mean (SD)	84.28(14.04)	81.27(12.23)	72.27(15.57)	0.01*	1 > 3**
Rbans					
Immediate Memory, mean (SD)	72.80(12.21)	75.28(12.18)	78.84(11.59)	0.209	
Visuospatial, mean (SD)	75.79(13.54)	82.20(14.39)	83.04(15.47)	0.170	
Attention, mean (SD)	96.76(12.54)	105.24(13.55)	102.72(17.86)	0.123	
Language, mean (SD)	79.28(11.78)	84.44(13.41)	84.64(13.47)	0.257	
Delayed memory, mean (SD)	75.56(14.85)	82.80(12.78)	85.71(19.40)	0.074	
Scale total scores, mean (SD)	73.64(9.29)	84.24(10.24)	82.72(11.28)	0.003**	1 < 2**; 1 < 3**

*CD-RISC* the Connor-Davidson Resilience Scale, *SDS* Self-rating depression scale, *SAS* Self-rating anxiety scale, *SSS* Somatic Self- rating Scale, *BIS-11* Barratt Impulsiveness Scale-11, *Rbans* The Repeatable Battery for the Assessment of Neuropsychological Status. Post-hoc pairwise comparisons (1 = Low resilience;2 = Medium resilience;3 = High resilience)

*p < 0.05

**p < 0.01

***p < 0.001

Table 2 revealed significant negative correlations among the dimensions of tenacity, strength, optimism and SDS scores. Additionally, the three dimensions of CD-RISC were also significantly negatively correlated with the BIS-11 scores. These significant differences all passed Bonferroni correction (*p* < 0.05/12).

**Table 2:** Inter-correlations among three dimensions of CD-RISC and SDS, SAS, BIS-11 and Rbans

	SDS	SAS	BIS-11	Rbans
Tenacity	−0.539*	−0.198	−0.279*	0.249
Strength	−0.620*	−0.234	−0.296*	0.340
Optimism	−0.543*	−0.210	− 0.242*	0.304

*SDS* Self-rating depression scale, *SAS* Self-rating anxiety scale, *BIS-11* Barratt Impulsiveness Scale-11, *Rbans* The Repeatable Battery for the Assessment of Neuropsychological Status

*Bonferroni corrected *p* < 0.05/12

Table 3 reported the factors which affect resilience during protracted METH withdrawal. And the results showed that the level of resilience was negatively associated with the level of depression (β = − 1.342, *P* < 0.001).

**Table 3:** Multiple linear regression for factors which affect resilience during protracted METH withdrawal

Variable	B	SE	B′	t	*P* value	95% Confidence Interval of B	
						Lower	Upper
Living styles	4.089	4.875	0.081	0.839	0.405	−5.644	13.821
Education	−1.073	4.058	−0.027	−0.265	0.792	−9.176	7.029
Age	0.204	0.456	0.048	0.448	0.656	−0.707	1.115
Duration of drug use	−0.582	0.390	−0.166	−1.493	0.140	−1.361	0.196
SDS	−1.342	0.270	−0.589	−4.963	< 0.001***	−1.882	−0.802
BIS-11	−0.034	0.184	−0.021	−0.184	0.855	−0.400	0.333
Rbans	0.371	0.218	0.170	1.697	0.094	−0.066	0.870

*B* Regression coefficient, *SE* Standard error, *B′* Standardized coefficient, *CI* Confidence interval

*SDS* Self-rating depression scale; *BIS-11* Barratt Impulsiveness Scale-11; *Rbans* The Repeatable Battery for the Assessment of Neuropsychological Status

R^2^ = 0.409; F = 6.512, *p* < 0.001

***p < 0.001

## Discussion

From what we know, this is the first study to investigate the relationships.

between resilience and impulsivity, cognitive function as well as emotion among METH-dependent patients during protracted METH withdrawal. In this study, we employed CD-RISC to measure resilience, which focuses on assessing different resilience factors (e.g. personality traits, beliefs) to maintain or regain mental health. And we evaluated the level of resilience based on a comprehensive analysis of resilience factors. We primarily found that high levels of resilience are associated with lower degrees of impulsivity and cognitive impairments and a lower level of depression. Early intervention to provide drug addicts with tools to encourage resilience is essential.

During early withdrawal, drug abusers will show a high frequency of impulsivity [43]. However, few studies have explored impulsive behaviors during the protracted withdrawal. The current study also found the connection between resilience and impulsivity during the protracted withdrawal. Although impulsivity may be a pre-existing characteristic that makes an individual prone to start taking drugs, it may also be a consequence of drug abuse [44, 45]. Previous METH use may cause changes in the brain, which in turn has a long-term impact on the changes within the typical decision-making process framework [46]. Thus, during the protracted withdrawal, METH users will show varying degrees of impulsivity. Many studies have investigated the relationship between resilience and impulsivity [47, 48]. The negative correlation between resilience and impulsivity in the period of protracted METH withdrawal may be explained by interconnections between specific neural circuits associated with resilience and impulsivity. What is more, the activation of ventromedial prefrontal cortex (vmPFC) has something to do with the promotion of resilience in response to stress [47]. Besides, impulsivity is inversely correlated to gray matter volume in the vmPFC [48, 49]. Thus, we inferred that long-term effect of METH abuse during the protracted withdrawal may result in abnormal activity or volume in vmPFC. High level of resilience may enhance the repair function of vmPFC and reduce the impulsivity [50]. In addition, impaired cognitive flexibility may have an adverse effect on problem-solving skills [51], coping with stress [52], and decision-making [15], which may increase impulsivity. However, individuals with high resilience are able to generate new strategies of action to reduce routine disturbances and enhance cognitive flexibility [53].

Cognitive functions will generally be impaired in patients addicted to drugs [54, 55]. The cognitive impairment will seriously affect the rehabilitation treatment for METH-dependent patients, especially during withdrawal. Therefore, it is necessary to find a way to improve cognitive deficit in METH dependence patients undergoing withdrawal. This study indicated that METH users with relative higher resilience had better cognitive function during protracted withdrawal. Understanding the impact of resilience on cognitive function impairment is relatively limited at this stage, and there is little direct evidence on their relationship. Despite this, the correlation can be broadly understood using the cognitive reserve hypothesis. Cognitive reserve is the ability to maintain cognitive function even if the brain injury occurs [56]. Harmonious relationship can improve cognitive reserve, and continuous positive stimulation can enhance the prevalent development of nerve tissue, promote new nerve pathways generation, and compensate for neurocognitive function impairment [57]. Generally, people with higher level of resilience can better deal with situations, creating a supportive psychological environment and promoting cognitive function. In contrast, people with low resilience levels cannot quickly restore balance when facing adverse events and exist in a state of chronic stress [58], such as protracted withdrawal. Chronic pressure can induce dendrites of the medial prefrontal cortex and hippocampal vertebral body neurons to retract, thereby causing damage to spatial memory and learning ability [59, 60]. Additionally, chronic stress can reduce brain-derived neurotrophic factor (BDNF) in the brain, affecting the memory processing by the hippocampus [61]. Charney indicated that the neurobiological reward system of people who are optimistic and hopeful in the context of extreme or chronic stress is either allergic or resistant to change [62]. Perhaps people with high resilience may also have highly functional emotional working memories that can maintain positivity and hope for the future even in the face of prolonged extreme stress and deprivation [29]. Furthermore, a meta-analysis suggested that there was a synergistic relationship between impulsivity and substance-related cognitive changes [63]. We inferred that the influence of resilience on cognitive function may be mediated by impulsivity, which may need further study to confirm.

What’s more, the relative higher resilience was associated with lower degree of depression during protracted withdrawal. And the three dimensions of CD-RISC (tenacity, strength and optimism) were all negatively related to SDS scores. The result was in line with previous research that resilience is correlated with the symptoms of depression [59] and high scores of mental resilience correspond to lower depressive symptoms [64]. However, the main underlying connection was still unknown. Even though, the possible mechanism can be explained as follows. Wang et al. found that resilience was negatively associated with perceived stress and depression [65]. And several studies suggested that perceived stress was positively correlated to depression in drug users [66, 67]. Perceived stress is defined as an individual’s cognitive assessment of their stress level [68], and effective stress management play a vital role in reducing the degree of depression in drug users [69]. In general, the perceived stress on drug users’ depression depends on their processing ability, while it is a coping ability, constantly adapting and rebounding in unfavorable environments. Compared with people with poor resilience, those with good resilience tend to feel less stress under challenging environments and are more likely to overcome stressful situations. Furthermore, when adjusted to a stressful environment, the symptoms of depression will naturally diminish [70]. Thus, it’s crucial to cultivate an individual’s resilience in order to better deal with the symptoms of depression caused by a stressful environment. Zhang et al. suggested that impulsivity is positively associated with depression during early METH withdrawal in Han Chinese population [71]. The craving for drugs is related to depression [72]. Similarly, high resilience may have positive effect on impulsivity and reduced the likelihood of caving for drugs, which may improve depressive symptoms.

Drug addiction is a complex process in which stress plays a crucial role [73]. Addiction and stress responses have a common neurobiological pathway that can be altered by environmental stressors [74]. Even after a long period of abstinence, it is easy to fall back into drug-seeking situations after a stressful experience [75]. Besides, the symptoms appeared in the withdrawal may add difficulties of intervention. Resilience refers to the relative protection of individuals against stress. Therefore, the current study postulated that improving resilience was necessary. For drug addicts, the barriers to resilience may include an imbalance between work and personal life, excessive exposure to stressful events, insufficient time and space to deal with negative emotions, and social isolation [76]. Effective interventions to promote resilience should ideally positively impact one or more of these barrier features, which may be available from the following three strategies [77]. Firstly, training, experience, and perception seem to be essential for enhancing resilience [78]. For example, METH addicts can learn “mindfulness”, which focuses on the present mental process, to avoid irrelevant, harmful external stimuli. Secondly, social support appears to be protective [79]. When social support is lacking, METH users may choose to escape and become muffled. Thus, establishing a correct concept of interpersonal communication is an excellent to obtain social support, because it is the basis for establishing good communication between people. Finally, effective coping styles may affect social adaptation in drug addicts, enabling them to become re-engaged with society [80]. For example, teaching stress management for drug addicts. Besides, this study found that the scores of SDS had a significant negative effect on resilience. Another way to increase an individual’s resilience level may be to improve the symptoms of depression. All in all, compulsory isolation of drug treatment centers can ensure that drug users are well-trained, thereby improving their adaptability and providing support designed to encourage better coping skills. Some combination of all or some of these strategies will increase the likelihood of more successful rehabilitation of METH-dependent users and may reduce the probability of relapse.

There are some limitations of this research. Firstly, the sample size is relatively small and the population is mainly concentrated in the eastern part of China. In the future, we will increase the samples and expand the scope to further validate our results. Secondly, it was a retrospective study and did not rule out recall errors and observer bias. Thirdly, the causality cannot be confirmed in this research. In this study, we found the relationship between resilience and the symptoms raised during protracted METH withdrawal. In the future, we will explore the direction of cause and effect. Fourthly, certain pre-abstinence factors (for example, early stressful life events) which might predict future recovery or deterioration were not evaluated [81]. Early stressful life events are related to mental health outcomes and may influence one’s resilience. Fourthly, only male METH-dependent patients were participants. It’s hard to say our findings also exist in female METH-dependent patients, so we will investigate female METH-dependent patients in the future. Finally, we also lack control group, it is hard to differentiate if some of the data are influenced by drug use. In future studies, we will include normal populations for further studies.

## Conclusion

The current study highlighted the role of resilience during protracted METH withdrawal. This study suggested higher resilience is correlated with lower impulsivity levels, better cognitive function and lower depression. For drug addicts, perhaps it may be effectively prevented and well cured by improving resilience.

## Supplementary Information

**Additional file 1: Supplemental Table 1.** Normality test of data. **Supplemental Table 2.** Homogeneity of Variances test. **Supplemental Table 3.** Tests of Between-Subjects Effects.

## Data Availability

The datasets generated and/or analysed during the current study are not publicly available due to the privacy of participants and because this is a part of the long-term study, but are available from the corresponding author on reasonable request.

## Abbreviations

METH: Methamphetamine
CD-RISC: The Connor-Davidson Resilience Scale
SDS: Self-rating depression scale
SAS: Self-rating anxiety scale
BIS-11: Barratt Impulsiveness Scale-11
Rbans: The Repeatable Battery for the Assessment of Neuropsychological Status
