# Supplementary material for: Effects of resilience on impulsivity, cognition and depression during protracted withdrawal among Chinese male methamphetamine users
Source: BMC Psychiatry. 2022 Jun 21;22:414. doi: 10.1186/s12888-022-04041-8 (PMC9215047; doi:10.1186/s12888-022-04041-8)
Supplement: Supplementary file 1 — Additional file 1: Supplemental Table 1. Normality test of data. Supplemental Table 2. Homogeneity of Variances test. Supplemental Table 3. Tests of Between-Subjects Effects. [file 12888_2022_4041_MOESM1_ESM.docx]

**Supplemental Table 1** Normality test of data.

|  | Shapiro-Wilk | | |
| --- | --- | --- | --- |
|  | Statistical magnitude | df | p value |
| CD-RISC | 0.971 | 112 | 0.078 |

Note: CD-RISC the Connor-Davidson Resilience Scale

**Supplemental Table 2** Homogeneity of Variances test.

|  | Levene Statistic | P |
| --- | --- | --- |
| SDS | 0.982 | 0.380 |
| SAS | 0.201 | 0.818 |
| BIS-11 | 1.241 | 0.295 |
| Rbans | 0.051 | 0.951 |

Note: SDS Self-rating depression scale; SAS Self-rating anxiety scale; BIS-11 Barratt Impulsiveness Scale-11; Rbans The Repeatable Battery for the Assessment of Neuropsychological Status.

**Supplemental Table 3** Tests of Between-Subjects Effects

|  | F | ηp^2^ | P |
| --- | --- | --- | --- |
| SDS | 21.736 | 0.380 | <0.001*** |
| SAS | 3.249 | 0.083 | 0.045* |
| BIS-11 | 4.968 | 0.121 | 0.010* |
| Rbans | 6.150 | 0.146 | 0.003** |

Note: SDS Self-rating depression scale; SAS Self-rating anxiety scale; BIS-11 Barratt Impulsiveness Scale-11; Rbans The Repeatable Battery for the Assessment of Neuropsychological Status. ηp^2^ partial eta squared. . *p < 0.05; **p < 0.01; ***p < 0.001.
